# Mechanical Properties of Additively Manufactured Composite Resin vs. Subtractively Manufactured Hybrid Ceramic Implant-Supported Permanent Crowns Before and After Thermal Aging

**DOI:** 10.3390/mi17010116

**Published:** 2026-01-16

**Authors:** Nilufer Ipek Sahin, Emre Tokar

**Affiliations:** 1 Health Sciences Institute, Gazi University, Cankaya, Ankara 06500, Turkey; nipeks@gmail.com; 2 Department of Prosthodontics, Faculty of Dentistry, Gazi University, Ankara 06510, Turkey

**Keywords:** 3D printing, additive manufacturing, CAD/CAM, dental crowns, fracture resistance, thermal aging, composite resins, polymer-infiltrated ceramic network

## Abstract

This study aims to compare the surface roughness and fracture resistance of implant-supported permanent crowns additively manufactured using composite resins (Crowntec, VarseoSmile) versus subtractively manufactured polymer-infiltrated hybrid ceramic (VITA Enamic) at various wall thicknesses using an experimental setup as close to clinical as possible. 180 crowns were fabricated in three thicknesses (1.0, 1.5, and 2.0 mm) and cemented onto titanium abutments. Experimental groups underwent thermal aging (10,000 cycles) to simulate one year of clinical service. Surface roughness was measured via profilometry, and fracture resistance was assessed using a universal testing machine. Composite resin crowns exhibited lower surface roughness and lower fracture resistance than subtractively manufactured crowns. No significant difference in fracture resistance was found between materials at 1.0 mm (*p* > 0.05). However, at 1.5 and 2.0 mm, hybrid ceramic network crowns showed significantly higher resistance (*p* < 0.01). It was concluded that, within the limitations of this 1-year simulated study, both material-method combinations met the biological threshold for surface roughness. Regarding fracture resistance, composite resins and hybrid ceramics satisfied clinical requirements for molar bite forces only at thicknesses of 1.5 mm and above. 1.0 mm thickness may pose a risk under high occlusal loads.

## 1. Introduction

The progressive increase in average life expectancy has led to a surge in the clinical application of dental implants and, subsequently, implant-supported prostheses [1]. Compared to other technical complications—such as abutment or screw loosening, loss of retention due to cement failure, and framework fractures—crown fractures remain the most frequent technical complication of implant-supported crowns over a five-year period [2]. Consequently, evaluating the mechanical strength of increasingly popular permanent crowns fabricated with contemporary materials and techniques is of paramount importance.

Computer-aided design/computer-aided manufacturing (CAD/CAM) techniques consist of additive manufacturing, also known as three-dimensional (3D) printing, and subtractive manufacturing, also known as milling [3]. Although both techniques are used in dentistry, CAD/CAM technology was considered more of a subtractive manufacturing technique until recently due to its widespread use [4].

Compared to subtractive manufacturing, additive manufacturing offers advantages such as higher accuracy, speed, better surface quality, cost-effectiveness, less waste material, and ease of intraoral repair. Despite these advantages, additive manufacturing has not been as widely used as subtractive manufacturing for producing permanent crown restorations until recently because of the lack of sufficiently durable and workable materials. In recent years, several 3D printable materials have been introduced to the market for permanent crown restorations, making it possible to take advantage of additive manufacturing to produce permanent restorations [5]. The routine use of implants to compensate for tooth loss has increased the importance of implant-supported crowns [6,7]. The development of composite resin materials suitable for additive manufacturing has contributed to the widespread use of 3D printers in the production of implant-supported permanent crowns.

Although numerous in vitro and in vivo studies have evaluated the physical performance of subtractively manufactured crowns—given their long history of clinical use—the number of studies assessing the fracture resistance of permanent restorations fabricated via 3D printing technology remains quite limited [2,3,8,9,10,11]. Among the few studies evaluating 3D-printed crowns, only two focused on implant-supported restorations: one investigating screw-retained and the other cement-retained crowns [10,11]. The only study conducted on implant-supported, cement-retained crowns, similar to our design, differed from our research in terms of crown morphology and the method of fracture load application [10]. Furthermore, we identified only one study that compared fracture resistance before and after artificial aging [11]. Collectively, this information highlights significant knowledge gaps regarding the comparative performance of additively versus subtractively manufactured crowns, particularly following artificial aging. According to the most recent reviews, the number of studies in the literature on the mechanical properties of permanent crowns produced with 3D printers using composite resin is currently limited [3,12]. All of the few studies evaluating surface roughness have been conducted on samples prepared in forms other than crowns, such as bars, disks and rectangular blocks [8,9,10,13]. To the best of the authors’ knowledge, this is the first study to evaluate the surface roughness of samples prepared in the form of crowns via additive manufacturing. The number of studies evaluating the fracture resistance of permanent crown restorations manufactured using 3D printing technology is also limited [5,9,11,14,15].

The objective of this study was to compare the fracture resistance and surface roughness of implant-supported permanent crowns fabricated via additive manufacturing using two different composite resin brands, Crowntec and VarseoSmile, and subtractive manufacturing (using a polymer-infiltrated ceramic network [PICN] material: VITA Enamic). In addition to benchmarking additive materials against the PICN reference, the study aimed to evaluate the mechanical performance differences between the two 3D-printing resins. The thermal aging technique was chosen to evaluate the effects of aging on these parameters because it is standardizable, widely accessible, and remains one of the most recognized and internationally accepted artificial aging procedures in the literature. Finally, the study investigated the influence of varying wall thicknesses to determine the optimal thickness for each material to withstand maximum occlusal chewing forces.

Studies evaluating the fracture resistance of crowns should be performed with an experimental setup that simulates the clinical conditions as much as possible to guide clinical practice. This study attempted to use an experimental setup as close to clinical conditions as possible, especially for fracture loading experiments, with the aim of simulating the worst-case scenario that could be encountered in the clinic.

The null hypotheses of the study were that (1) neither the manufacturing technique nor the material type would significantly affect the surface roughness or fracture resistance of the implant-supported crowns, and (2) artificial thermal aging simulating one year of clinical use would have no significant impact on the surface roughness or fracture resistance of the tested materials.

## 2. Materials and Methods

**Figure 1:**
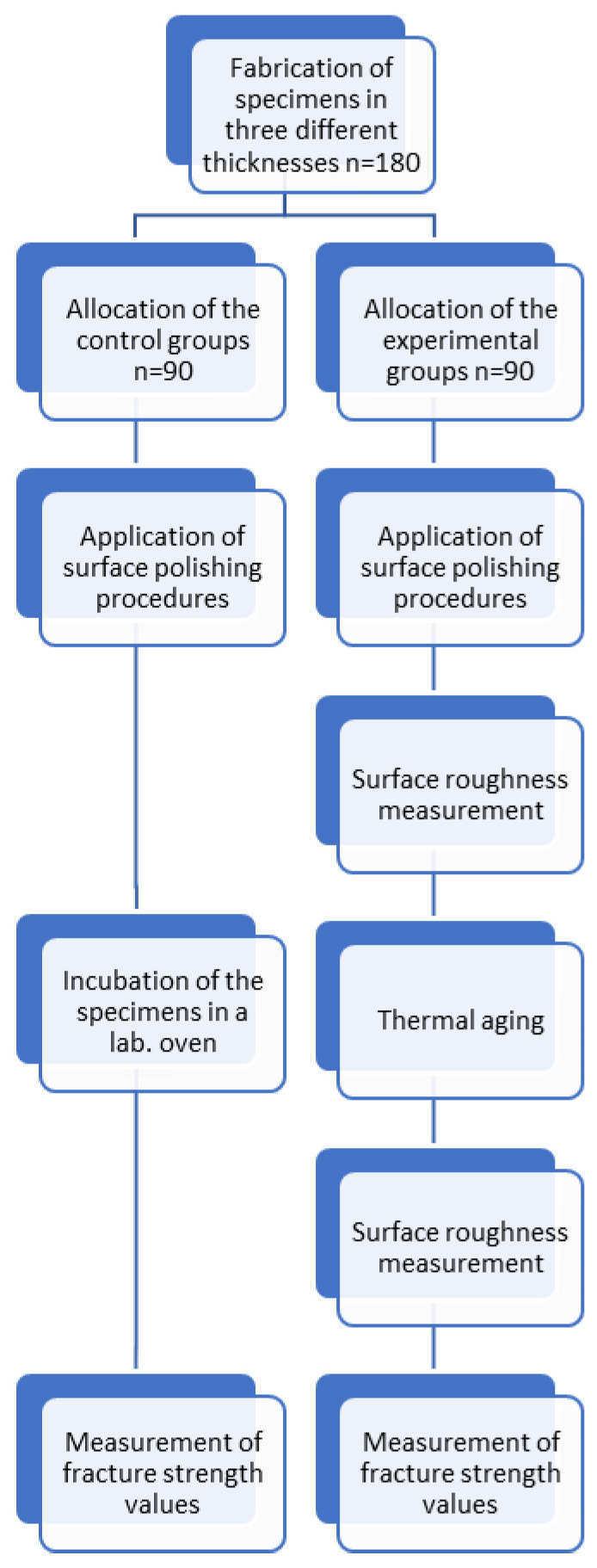
Schematic diagram showing study design.

The schematic diagram of the study design is shown in Figure 1.

### 2.1. Design and Preparation of Crown Samples

**Figure 2:**
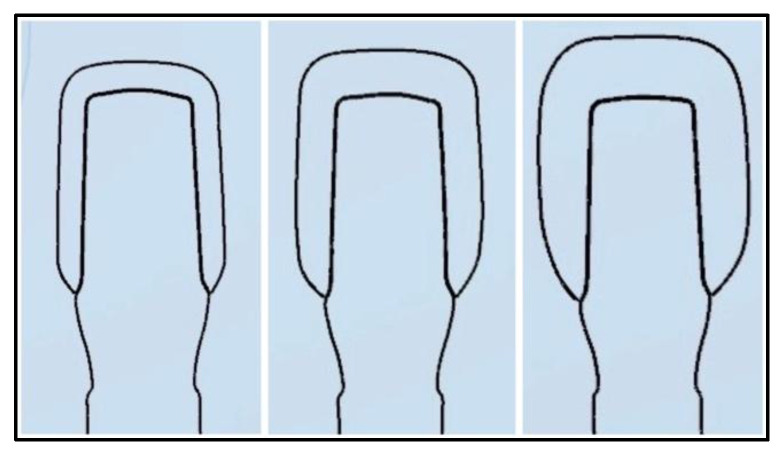
Cross-sectional CAD designs of crown restorations with wall thicknesses of 1.0, 1.5, and 2.0 mm.

**Figure 3:**
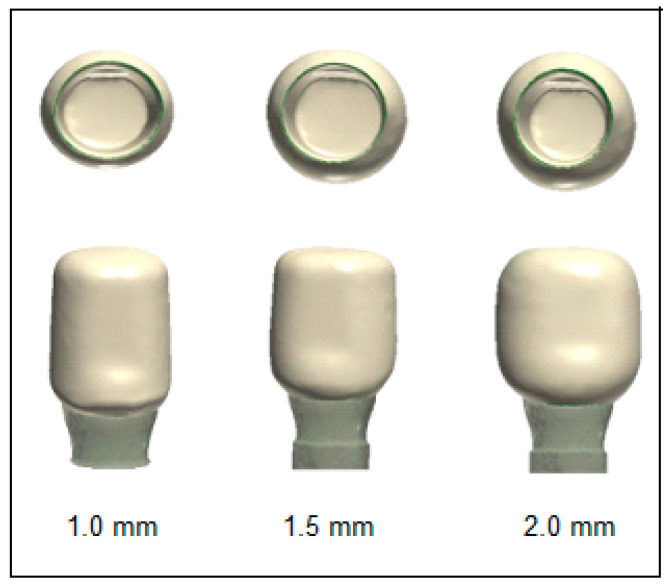
Images of designs created for different wall thicknesses using the 3Shape Dental Design 2021 design program.

Before crown design and manufacturing, implant analogs (PS IMPA 57821, BEGO, Bremen, Germany) were embedded in 15 mm × 15 mm × 20 mm polymethyl methacrylate (PMMA) based self-curing pink acrylic blocks. The abutments were screwed to implant analogs (PS TIA 57851, BEGO, Bremen, Germany) with a torque of 25 N/cm and retightened after ten minutes. One of the abutments was scanned using the 3Shape EScanner (3Shape, Copenhagen, Denmark), and the data obtained were transferred to the 3Shape Dental Design program (3Shape) for crown design (Figure 2). The crowns were designed with three different thicknesses, 1.0, 1.5, and 2.0 mm, with equal thicknesses of the occlusal, buccal, lingual, and proximal walls (Figure 3). Care was taken to ensure that the supporting structures were not located in the center of the occlusal region where the force would be applied during fracture testing. The cement space was set to 50 μm for each samples.

A total of 180 crowns were produced, 20 for each thickness in each material group. Ten of these 20 samples were designated the control, and 10 were designated the experimental group. The total sample size was determined by statistical power analysis using G*Power Software version 3.1 (effect size of 0.3, α = 0.05, observed power = 0.80).

Hybrid ceramic VITA Enamic blocks (VE, VITA Zahnfabrik; Bad Säckingen, Germany) were selected for subtractive manufacturing. A 5-axis milling CAD/CAM device (ImesCore 350i Loader, Eiterfeld, Germany) was used for subtractive manufacturing. The crowns were cleaned in an ultrasonic cleaner after their support was removed with a cutoff wheel.

To optimize the bond at the resin-metal interface—a critical factor for the longevity of the prosthesis—the internal surfaces of the CT and VS crowns, as well as the external surfaces of the abutments, were sandblasted with airborne-particle abrasion in strict accordance with the manufacturers’ instructions. For additive manufacturing, VarseoSmile Crown Plus (VS, Bego, Bremen, Germany) and Crowntec (CT, Saremco Dental AG, Rebstein, Switzerland) composite resins were utilized. DLP-based 3D printers, Varseo XS (Bego, Bremen, Germany) for VS, and MAX UV (Asiga, Sydney, Australia) for CT were used. The layer thickness was selected as 50 µm. The printing orientation was determined to be 0 degrees since it was reported that marginal adaptation is better than other build angles [16]. After production was completed, the CT crowns were cleaned with a 96% ethanol-soaked cloth, and the VS crowns were cleaned in an ultrasonic bath containing 96% ethanol. The crowns were cured in a polymerization device (Otoflash G171-6, NK Optik, Baierbrunn, Germany) with 2000 × 2 light exposures for CT and 1500 × 2 light exposures for VS according to the manufacturers’ recommendations. Brand names, manufacturers, and chemical compositions of the materials used in the study are listed in Table 1.

The inner surfaces of the CT and VS crowns were airborne-particle abraded (Zhermack Sand S24R, Marl, Germany) at 1.5 bar pressure with Al_2_O_3_ (Korox, BEGO, Bremen, Germany) from a distance of 10 mm for 10 s. The particle size of Al_2_O_3_ was 110 µm for CT, whereas it was 50 µm for VS. The outer surfaces of the abutments were airborne-particle abraded with the same sandblasting machine at 1.5 bar pressure with 50 µm particle-sized Al_2_O_3_ from a distance of 10 mm for 15 s. The VE group crowns were treated with 4.5% hydrofluoric acid (IPS Ceramic Etching Gel; Ivoclar AG, Schaan, Liechtenstein) for 60 s.

Surface polishing was applied to all the crowns. The Vita Enamic Polishing Set (VITA Zahnfabrik; Bad Säckingen, Germany) was used for VE and the Diacomp Twist composite polishing set (Eve Gmbh, Keltern, Germany) was used for CT and VS. Polishing of the restorations was finished using a cotton bur. The screw access holes of the abutments were sealed with polytetrafluoroethylene (PTFE) tape, Teflon. The PTFE tape was inserted into the access hole cavity and firmly compacted with an amalgam plugger until the hole was completely covered [17]. Silane (UltraDent Products GmbH, Cologne, Germany) and then a bonding agent (Gluma, Kulzer GmbH, Hanau, Germany) were applied to the inner surfaces of the crowns and the same operator cemented the crowns with finger pressure using dual-cure resin cement (Els Cem, Saremco Dental AG, Rebstein, Switzerland) [14,18]. The buccal, lingual, palatal and occlusal surfaces of the crowns were light-cured for 40 s to ensure polymerization.

### 2.2. Surface Roughness Measurements

To ensure the reproducibility of surface roughness measurements before and after aging, the crowns were first polished according to the manufacturer’s instructions. Subsequently, notches were created on the acrylic blocks to mark a consistent measurement line for each specimen. Before the thermal aging process, the surface roughness of the crowns in the experimental group was measured using a contact profilometer (Mahrsurf M300C, Mahr, Gottingen, Germany). The measurements were made with a measurement length of 1.75 mm and a speed of 0.5 mm/s. To obtain reliable results during the measurements, the device was calibrated with a reference calibration block after every 10 test sample measurements. The measurements were repeated 3 times. The surface roughness values were determined by calculating the arithmetic average of these three measurements.

### 2.3. Thermal Aging Process

Thermal aging was performed in a thermal cycler (SD Mechatronik Thermocycler, Julabo GmbH, FT 200, Seelbach, Germany) at 30 s intervals between 5 and 55 °C for 10,000 cycles, corresponding to one year of aging [19]. During the aging period, the control group samples were kept in distilled water in a heating cabinet (Kottermann Labortechnik, Uetze, Germany) at 37 °C.

After the thermal aging process was complete, the surface roughness measurements of the crowns in the experimental group were repeated 3 times with the same contact profilometer device from the locations where the first measurements were made, and the average Ra values of postthermal aging were calculated.

### 2.4. Fracture Resistance Measurements

**Figure 4:**
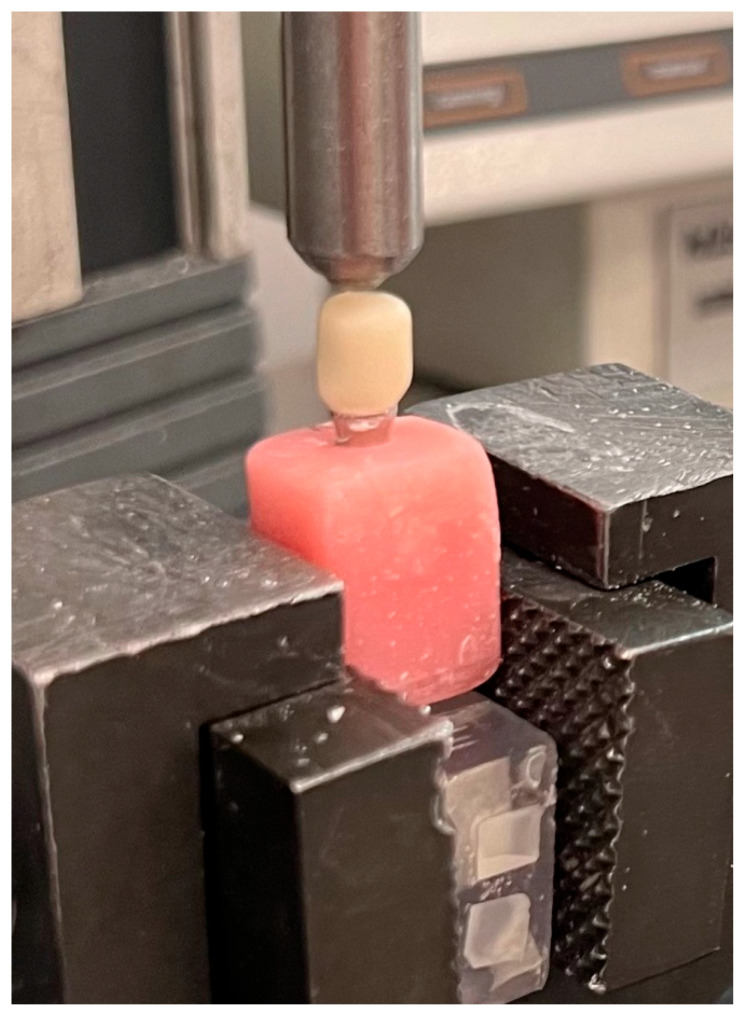
Measuring fracture resistance by a universal testing machine.

All the samples in the control and experimental groups were subjected to a fracture resistance test using a universal testing machine (Lloyd-LRX, Lloyd Instruments, Fareham, UK) (Figure 4). A spherical tip with a diameter of 5.0 mm was used during loading. Force was applied at a rate of 1 mm/min vertically to the center of each crown, corresponding to the point where the screw hole was located. The force application was continued until fracture occurred, and the fracture loads of the samples were recorded in Newtons by a computer program (Nexygen 4.0, Lloyd Instruments Ltd., Fareham, UK).

### 2.5. Statistical Analysis

Statistical analysis of the obtained data was performed with the SPSS 23.0 (SPSS Inc., Chicago, IL, USA) package program. The fracture resistance and surface roughness values were evaluated with the two-way ANOVA. Duncan’s post hoc test was used for the comparative evaluation of subgroups. Dependent samples *t*-tests were performed to assess the effect of thermal aging on surface roughness.

## 3. Results

### 3.1. Surface Roughness

The descriptive statistics of the surface roughness measurements and *t*-test results (*p* values) are presented in Table 2. A two-way ANOVA test indicated that the material type was a factor influencing surface roughness. Although no difference in surface roughness values was found between the crowns produced by additive manufacturing (VS and CT), the surface roughness values of crowns produced through subtractive manufacturing (VE) were found to be greater than those of VS and CT. Material thickness did not affect the surface roughness across all three material groups. Additionally, no statistically significant difference was observed in the roughness values obtained before and after thermal aging for all material and thickness groups (*p* > 0.05).

### 3.2. Fracture Resistance

The results of the fracture resistance measurements for the control and experimental groups are shown in Table 3.

**Figure 5:**
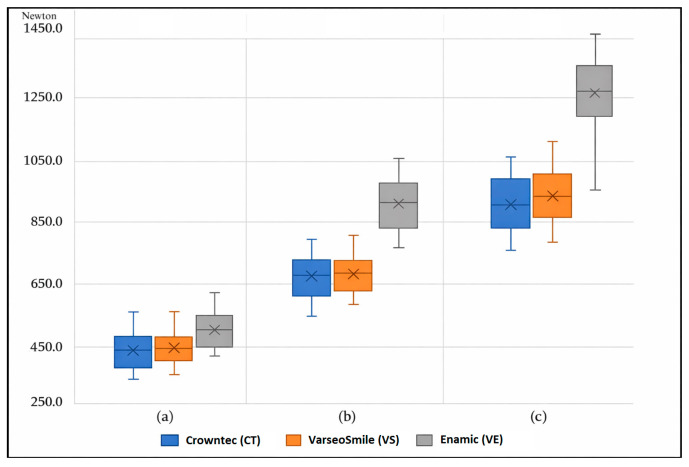
Fracture resistance of crowns with different thicknesses. (**a**) 1.0 mm, (**b**) 1.5 mm, (**c**) 2.0 mm.

The two-way ANOVA test results of fracture resistance indicated that the material type and wall thickness significantly differed (*p* < 0.01). In each material group, an increase in wall thickness resulted in a statistically significant difference in fracture resistance (*p* < 0.01). Thermal aging had no effect on fracture resistance (*p* > 0.05). In evaluating the wall thickness and material together, the comparative Duncan post hoc analysis revealed that there was no statistically significant difference in the fracture resistance values of the three materials at a thickness of 1.0 mm. It was also found that VE exhibited greater fracture resistance than VS and CT in 1.5 and 2.0 mm thicknesses (Table 4, Figure 5). No difference in fracture resistance was found between VS and CT for all three wall thicknesses.

## 4. Discussion

Surface roughness quantitatively describes the degree of unevenness or irregularities found on the surface of a material [3]. The increase in the surface roughness of crown restorations causes the increase in wear on the antagonist teeth and the adhesion of microorganisms and therefore results in stains, the formation of plaque, the loss of color stability, and soft tissue reaction, and so negatively affects the esthetic success and survival of the restoration [20]. Clinical studies have determined that 0.2 µm surface roughness of restorations in the mouth is the threshold value, especially in terms of bacterial plaque retention [21,22].

In this study, the surface roughness of the CT and VS groups was found to be significantly lower than that of the VE group. Although there were some differences between the groups, sufficiently smooth surfaces meeting the clinically accepted value (<0.2 µm) could be obtained for the crowns in all three material groups with the surface polishing process applied before the thermal aging process.

According to recent reviews, there is a limited number of studies investigating the surface roughness of permanent crowns additively manufactured with composite resins [3,12]. Most existing research has evaluated material surface roughness using geometries other than actual crown forms. For instance, one study compared the surface roughness of disks fabricated from two 3D-printable composite resins (CT and VS) and one resin nanoceramic used in subtractive manufacturing. While all three materials initially exhibited clinically unacceptable surface roughness, they generally reached acceptable levels following the polishing process [9]. Conversely, Bozoğulları et al. reported that the surface roughness of rectangular rod samples produced from CT was lower than that of those made from PICN (VE), a finding consistent with our study [13]. Furthermore, their study found no statistically significant difference in surface roughness before and after thermal aging, which also aligns with the results obtained in our research. In the three studies we encountered in the literature, thermal aging did not cause a statistically significant change in surface roughness values, as in our study [13,23,24].

According to the surface roughness results of this study, the null hypothesis stating that the manufacturing technique and material combination would have no effect on surface roughness was rejected. Conversely, the null hypothesis regarding the lack of effect of one-year thermal aging on surface roughness was accepted.

The mechanical resistance of prosthetic restorations against masticatory forces is one of the most important factors affecting the survival and clinical success of restorations. The maximum bite force is defined as “the maximum force that a person can reach while clenching their teeth without causing pain in the periodontal tissues” [25,26]. The maximum bite force reaches its highest value in the molar tooth region and decreases as it moves anteriorly, reaching 1/3 or even 1/4 of the maximum value at its lowest point [27]. In an in vivo study, the maximum bite force for molar teeth was found to be 490 N in men and 402 N in women, whereas in another study, it was found to be 522 N for men and 441 N for women [26,28]. In a study evaluating the situation for incisors, the maximum bite force was reported to be as low as 190 N in men and 50 N in women [29]. The results of some in vivo studies have shown that the maximum bite force to which implant-supported crowns are exposed is close to the maximum bite force exposed by natural teeth and is usually slightly lower [30,31,32].

According to the results obtained in this study (Table 2), no significant difference in fracture resistance was found among the experimental groups at a wall thickness of 1.0 mm (*p* > 0.05). However, at thicknesses of 1.5 and 2.0 mm, subtractively manufactured PICN crowns exhibited significantly higher resistance than additively manufactured composite resins (*p* < 0.01).

Based on the fracture resistance results, the null hypothesis that the manufacturing technique and material combination would have no effect on fracture resistance was rejected. Conversely, the null hypothesis stating that one-year thermal aging would not affect the fracture resistance of the crown materials was accepted.

The superior fracture resistance in the VE group is not solely due to the manufacturing process. Instead, these results should be interpreted by considering that the manufacturing technique and material properties were evaluated together as a combined variable. PICN materials feature a unique dual-network structure where a feldspathic ceramic network is infused with a polymer, effectively arresting crack propagation more efficiently than conventional composites [33]. Furthermore, while subtractive manufacturing utilizes industrially standardized, high-density blocks with minimal internal defects, additive manufacturing relies on a layer-by-layer deposition process [34], which can introduce interfacial weaknesses [35]. As demonstrated by these structural and methodological differences, previous reports have suggested that composite resins may be relatively weaker and that additive manufacturing possesses certain limitations compared to subtractive methods. However, our findings suggest that—likely due to recent advancements in both material quality and additive manufacturing techniques—implant-supported crowns produced via these digital workflows exhibit sufficient fracture resistance to meet clinical requirements. Despite the inherent advantages of the PICN microstructure, modern additively manufactured resins provide a safety margin well above physiological occlusal forces, even after the simulated aging process.

There are a limited number of studies in the literature comparing the fracture resistance values of crowns produced using subtractive and additive techniques [5,9,11,14,15]. According to the authors’ knowledge, 5 studies in the literature can be compared with this study in terms of fracture resistance. Two of these studies were performed on implant-supported crowns, and the other three used CAD/CAM-fabricated resin dies as abutments. Diken Türksayar et al. [15] evaluated the fracture resistance of screw-retained implant-supported crowns following thermomechanical aging and, consistent with our findings, reported that the VE group exhibited significantly higher fracture resistance than the VS and CT groups. Similarly, Dönmez and Okutan [14] evaluated cement-retained, anatomical implant-supported crowns with homogenous wall thicknesses ranging from 1.5 to 2.5 mm, but unlike our methodology, fracture resistance was measured without prior aging, and no significant differences were observed among the materials tested. In another study, Zimmermann et al. [11] used SLA-produced dies to evaluate various subtractive materials and a 3D-printed permanent resin after thermomechanical aging; similar to our protocol, they utilized wall thicknesses (0.5, 1.0, and 1.5 mm) and confirmed that fracture resistance increases with thickness, though in contrast to our findings, they reported that the 3D-printed material exhibited higher fracture resistance than VE at thicknesses of 1.0 and 1.5 mm. Furthermore, Suksuphan et al. [5] utilized 3D-printed dies to evaluate crown materials, including VE and VS, without prior aging; they confirmed that fracture resistance increases proportionally with thickness and that VE significantly outperforms VS, aligning with our results. Finally, Çakmak et al. [9] evaluated VE, VS, and CT crowns using anatomical forms and milled epoxy resin dies. Following mechanical aging, the VE group showed superior fracture resistance, consistent with our findings. However, in contrast to our study, they reported that VS exhibited higher fracture resistance than CT.

Notably, the results obtained in the studies we reached in the literature are not consistent with each other. The results of the study by Türksayar et al. are qualitatively similar to our study but they differ quantitatively [15]. To understand the source of differences among the results obtained in the studies we summarized above, it would be useful to briefly discuss the characteristics of the study setups used in the fracture resistance tests.

The main factors that can affect the results of studies evaluating the fracture resistance of crowns are the properties of the materials used in production, the position of the fracture load applied during the test [36], and the elastic modulus of the supporting structure (dies or abutments) [37,38]. Additionally, it is appropriate to include the crown form and wall thickness used in the experiment among the parameters that may influence the results. A fracture test setup is formed by the combination of all these parameters and factors and significantly affects the results obtained from the study. Unless the experimental setups used in such experiments are standardized, it will not be possible to objectively compare the results obtained from studies conducted with great effort and to derive information that will guide clinical applications from these results.

In the current study, an attempt was made to create an experimental setup as close as possible to clinical conditions. Crowns with homogeneous wall thickness were produced from materials routinely used in clinical practice, using devices and usage parameters in accordance with the recommendations of the material manufacturers.

In the fracture load tests, the loading force was applied to the center of the occlusal surfaces of the crowns, the point where the screw hole is located. Thus, the weakest point of the crown was selected, and the measurement was made according to the worst-case scenario. Our study is different from existing studies in some ways and, in our opinion, has an experimental setup that simulates clinical conditions as well as possible.

As limitations of this study, one year of aging did not cause a significant difference in terms of either roughness or fracture resistance. However, permanent restorations are expected to function for 10–15 years. Therefore, studies evaluating performance at 5-, 10-, and 15-year intervals are required to assess the long-term fatigue behavior and hydrolytic degradation of implant-supported composite resin crowns fabricated via additive manufacturing techniques, as well as those produced using other manufacturing technologies and materials. Although the fact that crowns with homogeneous thicknesses on each wall were preferred in our study rather than in the form of natural teeth may seem to be a limitation, this form was preferable for evaluating the effect of thickness on fracture resistance more precisely.

## 5. Conclusions

In light of the present study, the following conclusions emerge:

1. The implant-supported permanent crowns produced by additive manufacturing techniques using composite resin meet the clinical requirements regarding surface roughness and fracture resistance.
2. Although the wall thickness of implant-supported crowns does not affect surface roughness, fracture resistance increases proportionally with wall thickness. Based on these results, it can be concluded that a wall thickness of 1.5 mm or greater is sufficient for implant-supported crowns in the molar region for all three materials, whereas a wall thickness of 1.0 mm may be considered for use only in incisors when necessary.
3. One year of thermal aging has no effect on the fracture resistance and surface roughness of implant-supported permanent crown materials.

## Figures and Tables

**Table 1:** Brand names, manufacturers, and chemical compositions of the materials used in the study.

Material	Material Name	Material Composition	Manufacturer
Composite resin for additive manufacturing	VarseoSmile CrownPlus A2	Esterification products of ethoxylated 4,4′-isopropylidenediphenol and 2-methylprop-2-enoic acid; silanized dental glass, methyl benzoylformate, and diphenyl(2,4,6-trimethylbenzoyl)phosphine oxide. The inorganic filler content (particle size: 0.7 µm) is 30–50% by weight.	Bego Gmbh, Bremen, Germany
Composite resin for additive manufacturing	Crowntec A2	Esterification products of ethoxylated 4,4′-isopropylidenediphenol and 2-methylprop-2-enoic acid, silanized dental glass, pyrogenic silica, and initiators. The inorganic filler content (particle size: 0.7 µm) is 30–50% by weight.	Saremco Dental AG, Rebstein, Switzerland
Polymer-infiltrated ceramic network (PICN) material for subtractive manufacturing	Vita Enamic M2 HT	Polymer-Infiltrated Ceramic Network (PICN) material, composed of two interlocking networks. Ceramic Network (approx. 86 wt%/75 vol%): Fine-structure feldspathic ceramic (aluminum oxide enriched) with the following oxide components: Silica, Alumina, Sodium oxide, Potassium oxide, Boron trioxide, Zirconia, Calcium oxide Polymer Network (approx. 14 wt%/25 vol%): Cross-linked polymer structure infiltrated into the ceramic pores: UDMA (Urethane Dimethacrylate) TEGDMA (Triethylene Glycol Dimethacrylate)	Vita Zahnfabrick, Bad Säckingen, Germany

**Table 2:** Descriptive statistics of the surface roughness measurements (Ra, µm) and *t*-test results (*p* values).

		Before Thermal Aging		After Thermal Aging		*p* Value
Group	mm	Mean ± SD	Median (Min–Max)	Mean ± SD	Median (Min–Max)	
CT	1.0	0.141 ± 0.038	0.126 (0.098–0.214)	0.138 ± 0.036	0.138 (0.090–0.199)	0.699
	1.5	0.146 ± 0.031	0.144 (0.102–0.198)	0.142 ± 0.036	0.136 (0.090–0.207)	0.579
	2.0	0.136 ± 0.026	0.136 (0.089–0.183)	0.137 ± 0.023	0.141 (0.101–0.179)	0.736
VS	1.0	0.147 ± 0.038	0.138 (0.105–0.225)	0.148 ± 0.039	0.145 (0.085–0.222)	0.879
	1.5	0.137 ± 0.028	0.137 (0.098–0.175)	0.142 ± 0.028	0.144 (0.103–0.189)	0.111
	2.0	0.135 ± 0.027	0.133 (0.092–0.177)	0.127 ± 0.029	0.117 (0.093–0.169)	0.116
VE	1.0	0.166 ± 0.015	0.167 (0.144–0.191)	0.168 ± 0.014	0.170 (0.145–0.195)	0.324
	1.5	0.178 ± 0.018	0.171 (0.155–0.214)	0.182 ± 0.022	0.178 (0.156–0.216)	0.375
	2.0	0.176 ± 0.021	0.175 (0.144–0.210)	0.173 ± 0.021	0.168 (0.146–0.211)	0.226

SD: Standard Deviation CT: Crowntec VS: VarseoSmile Crown Plus VE: Enamic.

**Table 3:** Descriptive statistics of the fracture resistance measurements (N).

		Control Group		Experimental Group	
Group	mm	Mean ± SD	Median (Min–Max)	Mean ± SD	Median (Min–Max)
	1.0	425.2 ± 66.3	444.1 (326.1–542.6)	413.8 ± 76.7	395.6 (321.3–553.5)
CT	1.5	670.7 ± 74.8	676.7 (563.0–791.8)	657.1 ± 70.8	665.9 (513.3–736.9)
	2.0	909.7 ± 97.4	892.5 (781.6–1063.2)	902.4 ± 100.3	912.0 (756.5–1051.8)
	1.0	444.1 ± 82.9	420.5 (349.8–622.8)	434.3 ± 72	423.1 (328.5–592.2)
VS	1.5	688.3 ± 70.8	687.3 (584.0–841.7)	676.4 ± 69.4	679.4 (578.8–794.3)
	2.0	953.1 ± 89.8	939.5 (824.5–1109.1)	935.2 ± 92.9	939.2 (782.3–1059.2)
	1.0	503.4 ± 51.5	506.0 (425.6–613.4)	480.5 ± 65.1	459.3 (413.6–574.7)
VE	1.5	930.7 ± 83.6	937.6 (806.1–1063.1)	898.4 ± 92.5	914.0 (782.8–1012.7)
	2.0	1291.3 ± 127.8	1296.4 (1042.7–1503.2)	1245.2 ± 144.9	1229.2 (968.5–1447.1)

SD: Standard Deviation CT: Crowntec VS: VarseoSmile Crown Plus VE: Enamic.

**Table 4:** Duncan post hoc analysis results of the fracture resistance values of the experimental and control groups (N).

mm	CT	VS	VE
1.00 mm	419.50 ± 70.00 ^d^	439.20 ± 75.76 ^d^	491.90 ± 58.29 ^d^
1.50 mm	663.90 ± 71.21 ^c^	682.30 ± 68.51 ^c^	914.50 ± 87.37 ^b^
2.00 mm	906.00 ± 96.30 ^b^	944.10 ± 89.40 ^b^	1268.00 ± 135.07 ^a^

Rows and columns with the same letter indicate that the difference is not statistically significant. CT: Crowntec VS: VarseoSmile Crown Plus VE: Enamic.

## Data Availability

The data that support the findings of this study are available from the corresponding author upon reasonable request.

## Abbreviations

The following abbreviations are used in this manuscript:

3D	Three-dimensional
CAD/CAM	Computer-Aided Design/Computer-Aided Manufacturing
CT	Saremco Crowntec
PMMA	Polymethyl methacrylate
PTFE	Polytetrafluoroethylene
VE	Vita Enamic
VS	VarseoSmile Crown Plus
